# Genome-wide mapping of Hif-1α binding sites in zebrafish

**DOI:** 10.1186/s12864-015-2169-x

**Published:** 2015-11-11

**Authors:** David Greenald, Justin Jeyakani, Bernd Pelster, Ian Sealy, Sinnakaruppan Mathavan, Fredericus J. van Eeden

**Affiliations:** Bateson Centre, Department of Biomedical Science, The University of Sheffield, Western Bank, Sheffield, UK; Lee Kong Chian School of Medicine, Nanyang Technological University, 11 Mandalay Road, Singapore, 308232 Singapore; The Genome Institute of Singapore, Biopolis, Biopolis Street, Singapore, Singapore; Institute of Zoology, University of Innsbruck, Technikerstr, Innsbruck, Austria; Wellcome Trust Sanger Institute, Wellcome Trust Genome Campus, Hinxton, UK

**Keywords:** Hypoxia, ChIP-seq, zebrafish, von Hippel-Lindau, Microarray, Hypoxia Response Element, Hif-1α

## Abstract

**Background:**

Hypoxia Inducible Factor (HIF) regulates a cascade of transcriptional events in response to decreased oxygenation, acting from the cellular to the physiological level. This response is evolutionarily conserved, allowing the use of zebrafish (D*anio rerio)* as a model for studying the hypoxic response. Activation of the hypoxic response can be achieved in zebrafish by homozygous null mutation of the von Hippel-Lindau (*vhl*) tumour suppressor gene. Previous work from our lab has focused on the phenotypic characterisation of this mutant, establishing the links between *vhl* mutation, the hypoxic response and cancer. To further develop fish as a model for studying hypoxic signalling, we examine the transcriptional profile of the *vhl* mutant with respect to Hif-1α. As our approach uses embryos consisting of many cell types, it has the potential to uncover additional HIF regulated genes that have escaped detection in analogous mammalian cell culture studies.

**Results:**

We performed high-density oligonucleotide microarray analysis of the gene expression changes in von Hippel-Lindau mutant zebrafish, which identified up-regulation of well-known hypoxia response genes and down-regulation of genes primarily involved in lipid processing. To identify the dependency of these transcriptional changes on HIF, we undertook Chromatin Immunoprecipitation linked next generation sequencing (ChIP-seq) for the transcription factor Hypoxia Inducible Factor 1α (HIF-1α). We identified HIF-1α binding sites across the genome, with binding sites showing enrichment for an RCGTG motif, showing conservation with the mammalian hypoxia response element.

**Conclusions:**

Transcriptome analysis of *vhl* mutant embryos detected activation of key hypoxia response genes seen in human cell models of hypoxia, but also suppression of many genes primarily involved in lipid processing. ChIP-seq analysis of Hif-1α binding sites unveiled an unprecedented number of loci, with a high proportion containing a canonical hypoxia response element. Whether these sites are functional remains unknown, nevertheless their frequent location near transcriptional start sites suggests functionality, and will allow for investigation into the potential hypoxic regulation of genes in their vicinity. We expect that our data will be an excellent starting point for analysis of both fish and mammalian gene regulation by HIF.

**Electronic supplementary material:**

The online version of this article (doi:10.1186/s12864-015-2169-x) contains supplementary material, which is available to authorized users.

## Background

Most multicellular life is heavily dependent on oxygen for survival. Multicellular organisms inhabit environments that have changing oxygen availability, and show conserved mechanisms to cope with extremes of oxygen availability which can occur both in their environment and within their tissues [1]. This high level of conservation means that non-mammalian model organisms can be employed to study the regulation of the hypoxic response. Furthermore, studies in fish might also provide the basis to understand how such responses are modified in aquatic environments, where oxygen availability can widely vary [2].

Hypoxia Inducible Factor (HIF) is central to the response to the lack of oxygen in the environment [3]. In order to maintain homeostasis; cells, tissues and organisms adapt to first cope with the lack of oxygen and then act to redress the underlying cause [3]. In order to reduce oxygen consumption, the HIF transcription factor orchestrates a metabolic shift from aerobic metabolism to glycolysis. It leads to: 1) Alteration in the composition of the electron transport chain [4]. 2) Induction of pyruvate dehydrogenase kinase switching metabolism away from oxidative phosphorylation [5, 6]. 3) Up-regulation of glycolytic genes [7, 8]. 4) Regulation of mitochondrial turnover [9, 10]. The HIF pathway also increases glucose trafficking into the cell by up-regulation of the glucose transporter *GLUT1* [11]. To counteract hypoxia, HIF stimulates erythrocyte production through up-regulation of *EPO* and a host of iron absorption related genes [12–16], and by increasing angiogenesis through increased production of angiogenic factors, like VEGF [17–20]. More recently, HIF has been implicated in lipid processing, mice carrying a hepatic knockout of *Vhl* develop severe hepatic steatosis with impaired fatty acid oxidation, decreased lipogenic gene expression, and increased lipid storage capacity [21]. Activation of the HIF pathway is implicated in tumour development and growth, as the interior of most solid tumours is hypoxic. Furthermore loss-of-heterozygosity of *VHL* leads to activation of HIFs, which is essential for VHL-driven tumorigenesis [22–24].

The HIF transcription factor is a basic-helix-loop-helix heterodimer consisting of an αand β subunit [25, 26]. The expression of HIF-α is regulated by the Prolyl-Hydroxylase Domain containing enzymes (PHD 1–3), which act to hydroxylate HIF-α under conditions of plentiful oxygen, leading to its recognition by von Hippel Lindau (pVHL) and subsequent proteosomal degradation [27–31]. Thus under conditions of normoxia, HIF-α is continually degraded. Under hypoxic conditions or, for instance, after mutation of VHL, the breakdown of HIF-α is inhibited. This leads to its translocation into the nucleus to bind HIF-β, creating the HIF dimer. This dimer is then capable of binding to Hypoxia Response Elements (HRE) within the genome which leads to transcriptional activation of target genes [12, 32]. HRE’s are characterised by a RCGTG binding motif, functional motifs are often found in the promoters of hypoxia response genes but have also been seen to act distally [33]. Previous work from the Ratcliffe and Mole labs have used the MCF-7 breast cancer cell line, stimulated using the hydroxylase inhibitor dimethyloxalylglycine (DMOG) or ≤1 % oxygen for 16 h in order activate the HIF response. These screens identified binding sites for both HIF-1α and HIF-2α binding sites using ChIP-chip [34] and ChIP-seq [33]. Both studies identify the canonical HRE, with Mole et al., finding 546 HIF-1α binding sites (putatively linked to 394 loci, 20.8 % of which are up-regulated FC ≥ 4, 15.6 % down-regulated) and 143 HIF-2α binding sites (134 loci, 32.8 % up-regulated FC ≥ 4, 1.5 % down-regulated). Similarly, the Schödel study found 400 HIF-1α binding sites (356 loci) and 425 HIF-2α binding sites (357 loci) [33]. Additionally, Gene Set Enrichment Analysis found strong correlation between HIF binding sites and up-regulation of genes, whilst there was no correlation between down-regulated genes [33]. Contrary to the situation humans, in zebrafish, few functional HREs have been defined. The HREs which likely control *erythropoietin, igfbp1a* and *period1b* expression in zebrafish have been identified, showing an identical motif to human [35–37]. The HREs for four Hif-2α specific genes, *birc5a/b* and *leg1a/b* have been identified [38, 39]. Thus, in order to further develop the zebrafish as a model for research into hypoxic signalling, there is scope for systematic identification HIF binding sites.

Heterozygous mutation of *VHL* in humans predisposes affected individuals to the development of highly vascularised tumours and cysts upon loss of heterozygosity of the remaining *VHL* allele, this is known as VHL disease [40]. In order to study this disease further, the *vhl* mutant zebrafish line was created. Homozygous mutants display a systemic hypoxic response that occurs once the remaining maternal *vhl m*RNA is depleted [41]. The earliest activation of the Hif pathway can be seen by 24 h post fertilisation (hpf) with up-regulation of *phd3 (egln3), ndufa4* and *ldha1a* detectable by *in situ* hybridisation [42]. Activation of the hypoxic response manifests itself in increased *vegf* receptor-dependent increases in neovascularisation [42]. In addition, *vhl* mutants exhibit strong increases in both red and white blood cell lineages, reminiscent of Chuvash polycythemia. This is a human disease caused by particular *VHL* mutations [42, 43]. Zebrafish *vhl* mutants display pronephric abnormalities from soon after the nascent kidney becomes functional., Interestingly, the cells of the pronephros display a phenotype reminiscent of the clear cell phenotype seen in clear cell Renal Cell Carcinoma (ccRCC), a disease caused by loss of heterozygosity of *VHL,* but do not develop tumours or cysts [42, 44, 45]. We chose the *vhl* mutant line as a model for hypoxia as we believed that genetic inactivation of *vhl* offered a stronger and more stable activation of the Hif pathway than pharmacological activation or physical hypoxia, whilst avoiding complications due to developmental retardation and other abnormalities resulting secondarily from severe hypoxia.

Here we report analysis of the gene expression changes in *vhl* mutant embryos, finding differential regulation of both key hypoxia response genes and genes not commonly associated with mutation of *vhl*. We report analysis of the Hif-1α binding sites within both *vhl* mutant and wild-type samples, finding a greater number binding sites than expected from mammalian studies [39, 40], and identify a HRE motif in zebrafish.

## Results

### Identification of genes differentially expressed in *vhl* mutant zebrafish

*Vhl* mutant zebrafish has been previously shown to exhibit a systemic hypoxic response with increased levels of Hif-1α seen in *vhl* deficient tumours [41, 42, 46]. Here we perform one-colour microarray analysis using an Agilent platform to determine the changes in gene expression caused by homozygous mutation of *vhl* at 4dpf. We chose to perform experiments at 4dpf because the phenotype of *vhl* mutants is clearly developed by 4dpf but embryos still appear healthy, without oedema. Therefore, using this time point may reduce the number of gene expression changes associated with secondary complications when compared to a later stage of development. For both wild-type and *vhl* mutant conditions, three biological replicates were created, with each replicate consisting of material from 30 embryos. *Vhl* samples were compared against wild-type samples using limma analysis for single channel microarray data utilising background correction and quantile data normalisation [47–50]. A *p*-value of ≤0.01 was used as a cut-off for significance and entities with a fold-change of greater or equal to two were considered to be differentially regulated. This produced a probeset of 1409 differentially regulated entities, of these, 1022 had unique Unigene ID’s, which corresponded to 737 genes with unique Unigene description. Of these genes, 295 were up-regulated and 442 were down-regulated (Additional file 1). The complete list of expressed entities can be found in Additional file 2.

The transcriptional response to hypoxia has been well studied in mammalian cells and zebrafish have been used as a model to study this pathway [33, 41, 42, 46, 51, 52]. In our microarray we detected significant up-regulation of an array of hypoxia response genes in *vhl* mutants. We detect increased expression of genes that regulate the HIF pathway itself, such as Egl nine homolog 3 *(C. elegans*) (*egln3 (*aka *phd3)*) which our lab has utilised as a live hypoxia reporter [46]. Similarly, we see an increase in classical hypoxia response genes such as, the key regulator of angiogenesis Vascular endothelial growth factor Ab (*vegfab*) [53]. A number of glycolytic genes were also significantly up-regulated; 6-phosphofructo-2-kinase/fructose-2,6-biphosphatase 3 (*pfkfb3*) [8], and Lactate dehydrogenase A4 (*ldha*) [7]. Additionally, a number of genes relating to processing of the extracellular matrix were up-regulated, including; Procollagen-lysine 1, 2-oxoglutarate 5-dioxygenase 1a (*plod1a*) [54] and Procollagen-proline, 2-oxoglutarate 4-dioxygenase (proline 4-hydroxylase), alpha polypeptide 2 (*p4ha2*) [55]. Interestingly, we see a large number of down-regulated genes. Such transcriptional repression is not commonly associated with activation of the HIF response in cellular models [33].

These gene expression changes in *vhl* mutants highlighted above were reflected in the results of Gene Ontology (GO) Term based analysis performed using Database for Annotation, Visualization and Integrated Discovery (DAVID) [56, 57]. The complete entity lists of differentially regulated genes in *vhl* mutants (found in Additional file 1) were uploaded to DAVID using Unigene ID as the identifier and analysed on the basis of the Biological Processes that they are involved in. Enrichment of biological processes for up-regulated transcripts found key terms associated with the hypoxic response such as; “oxygen transport”, “glycolysis” and “hemopoiesis”, strongly implicating activation of the HIF-pathway [2, 3]. Down-regulated transcripts were enriched in a number of Biological Processes associated with lipid processing such as; “lipid biosynthetic process” and “fatty acid metabolic process”. The complete list of enriched biological processes for both up- and down-regulated transcripts can be found in Additional file 3.

In order to validate of our microarray results, quantification of fold-changes by quantitative polymerase chain reaction (q-PCR) was performed using the Pfaffl method [58] and the correlation between these and the microarray fold changes was assessed using Spearman’s Rho. q-PCR was performed for 24 up-regulated genes with normalisation against the reference genes *18 s* and *wnt5a*, both of which have been used previously as reference genes for quantification of mRNA in zebrafish [59, 60]. Correlation was significant between the fold-changes detected by microarray and by q-PCR analysis regardless of which reference gene was used for the qPCR analysis (18 s: ρ = 0.7496, *p* < 0.001, *n* = 24; wnt5a: ρ = 0.7567, *p* < 0.001, *n* = 24), an observation that is in line with results from other studies [61].

### ChIP-qPCR experiments show specific enrichment for the *epo*-HRE in *vhl* mutants

**Fig. 1:**
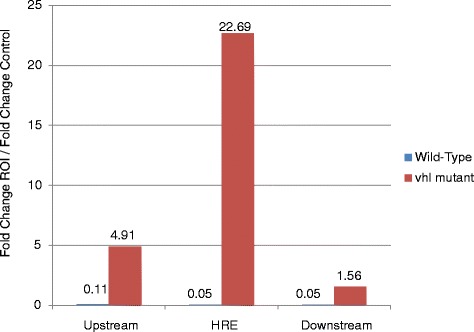
*vhl* mutants shows specific enrichment for the *epo-*HRE. Graph showing the result of a ChIP-qPCR experiment. In *vhl* mutants HIF is stabilised and is expected to bind to HREs. Strong enrichment (22-fold) for the region spanning the *epo-*HRE is seen in *vhl* mutants, whereas the wild-type HRE, and flanking regions both up- and down-stream of the HRE are much less enriched. A control region ~2Kb distally was used for comparison. Details for the sequences and relative positions of primers as well as the fold change calculation can be found in the Additional file 14 and the Methods section

The HIF binding consensus sequence, the Hypoxia Response Element, has been widely studied in mammalian cells; from its initial identification due to its association with transcriptional activation of erythropoietin, to extensive ChIP-sequencing of cells exposed to hypoxic conditions [12, 33]. In fish, a functional HRE that influences the expression of erythropoietin has been identified, with the consensus sequence being identical to that found in mammals (ACGTGCTG) [35]. We tested several custom-made/commercial Hif-1α antibodies for their suitability for ChIP. The first step was to demonstrate that the Hif-1α antibody was able to specifically bind to the epo-HRE. This was achieved by performing ChIP followed by q-PCR for the region surrounding the previously identified HRE [35]. The DNA for this was isolated from a group of 2400 wild-type larvae pooled into a single sample and 2400 *vhl* mutant larvae pooled into a separate sample, this DNA was later used for the ChIP-seq experiments. Enrichment was measured using a variation on the Pfaffl method [58], where the CT value for both the ChIP and the input control are calculated for the region of interest and additionally for a distal control region. We would expect that in *vhl* mutants, there would be greater enrichment for the region directly over the HRE than for regions that are both up- and down-stream to it, whereas the enrichment would not be seen in wild-type ChIP material. Only one antiserum gave a positive result (Fig. 1): a polyclonal fish-specific Hif-1α antibody used previously to identify Hif-1α in hypoxia-treated zebrafish [62] This serum also gives good signals in western blots of larval zebrafish [46]. Therefore, this antiserum was used for the ChIP-seq experiments.

### Identification of Hif-1α binding sites in the zebrafish genome

As few Hif-1α binding sites have been identified in zebrafish and ChIP-sequencing has emerged as an appropriate technology for genome-wide DNA binding site analysis in zebrafish, we decided to sequence the ChIP material isolated from both wild-type and *vhl* mutants using one biological replicate of 2400 embryos for each. ChIP material was prepared for library submission using the Tru-Seq low input library preparation kit, (despite the use of large numbers of embryos only a relatively small amount of ChIP material could be isolated) and then sequenced using the Illumina platform. The sequencing data was analysed using Model-based analysis of ChIP-Seq (MACS) (version 1.4.2 20120305) which was able to call the number of peaks enriched in the ChIP material for *vhl* and wild-type with a *p*-value of ≤ 1x10^−5^ (corresponding to a score of >50 in MACS). In total, there were 1280 peaks for wild-type and 5177 peaks for *vhl* with a *p*-value of ≤10^−5^. Of 1280 peaks found in the wild-type sample, 157 peaks overlapped the 5177 peaks found in the *vhl* sample, equating to 12.3 %. Notably, wild-type peaks are shorter on average (114 bp versus 609 bp), and on a number of occasions multiple wild-type peaks fall within a single *vhl* peak. When the number of tags was examined, the 5177 *vhl* peaks represented 360746 tags and the 1280 wild-type peaks represented 12158 tags.

**Fig. 2:**
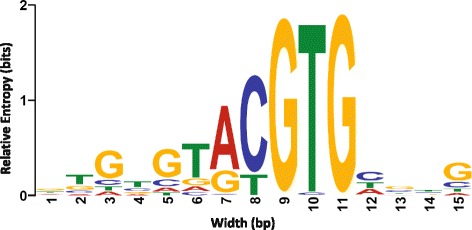
The primary motif found in *vhl* mutant ChIP-peaks. The most significant 1000 peaks from the sequencing of the vhl mutant ChIP material were analysed for the presence of sequence motifs in the 100 bp surrounding the centre of the peak using MEME. This identified a motif that closely resembles the known HRE consensus [41]. The y-axis shows the Relative Entropy in bits, a measure of the probability that the letter will be found at that position relative to the total information content of the stack, the x-axis shows the width of the motif in base pairs. More information can be found on the MEME website (http://meme-suite.org/doc/examples/memechip_example_output_files/index.html?man_type=web)

**Fig. 3:**
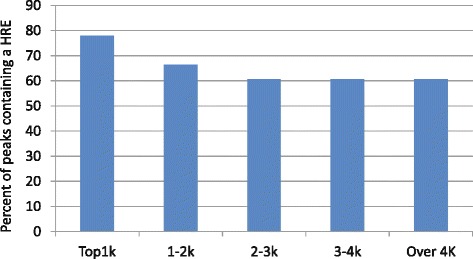
HREs are enriched in the most significant peaks. The peaks from our HIF ChIP-seq experiment were ranked according to *p*-value, a measure of their height above the (local) background of mapped sequence tags. They were then binned into groups of 1000, the percentage of peaks containing an HRE was then calculated, this showed that the most significant peaks show enrichment for HRE’s

**Fig. 4:**
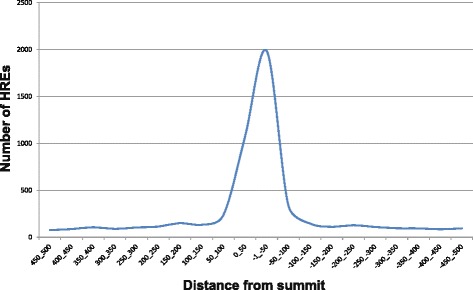
The RCGTG/HRE motif is enriched in regions in close proximity to the peak summit. The position relative to the peak summit of the total number of HRE’s in the *vhl* mutant data set was assessed. The X axis displays the distance from the summit in basepairs, and the y-axis the number of HREs that fall into a given window. The majority of peaks are found with ±100 bp of the summit when the surrounding 1 kb was analysed

In order to perform an unbiased identification of Hif-1α binding sites, *de novo* motif analysis was carried out using Multiple Em for Motif Elicitation (MEME) (version 4.9.0, http://meme-suite.org/) using ±50 bp from the summits of the top 1000 peaks from the vhl mutant data set [63]. This identified an RCGTG motif that is also observed in human HREs (Fig. 2) [33]. Similar analysis using the wild-type data set did not find any motifs. The RCGTG motif was then used to search against both the wild-type and vhl mutant data sets. We searched for the RCGTG motif in 100 bp surrounding the summit. This analysis identified 168 unique peaks in the wild-type data set and 4232 unique peaks in *vhl* data set [64]. Notably, of the 168 wild-type HRE peaks, 66 were found to overlap more than 50 % with the *vhl* peaks. When analysed using Motif Occurrence Detection Suite (MOODS) (v 1.0.1, http://www.cs.helsinki.fi/group/pssmfind/) with an e-value cut-off of 0.001 and Zv9 genome as a background, there was some degree of correlation between the peak *p*-value and presence of the RCGTG motif when peaks were ordered by *p*-value and binned into groups of 1000 (Fig. 3). Importantly, when the distribution of the RCGTG motif within the surrounding 1000 base pairs of the peak was analysed, a strong preference for the motif to be found close to the summit of the peak was observed (Fig. 4). This is important as the RCGTG motif is over represented in the genome, with it occurring at greater than one in every 256 bp by chance.

**Fig. 5:**
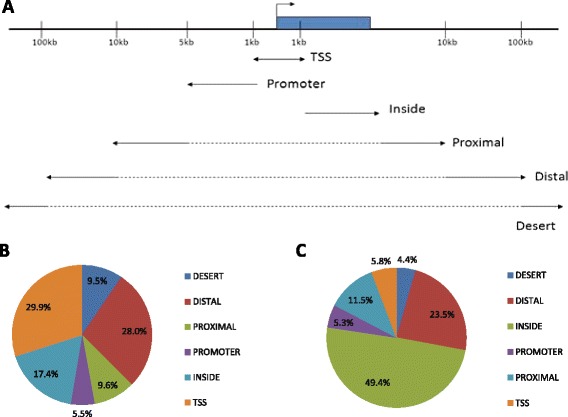
The distribution of Hif-1α binding sites in *vhl* mutant zebrafish. **a**: Definitions of genomic regions. TSS: If peak summit is 1Kb up or down-stream of the TSS of the closest gene (−1Kb to +1Kb). Promoter: From -5Kb upstream to upstream limit of the TSS (−1Kb). Inside: Inside the gene, including introns and exons but excluding areas covered by the TSS. Proximal: 5 Kb Up- and down-stream of the limits of the promoter and inside region. Distal: 90Kb up- and down-stream of the proximal regions. Desert: Any region up- or down-stream of the distal regions. **b**: The proportion of peaks containing HRE’s that fall within a given region for *vhl* mutants. **c**: The proportions of the genome that are described by the regions defined in **a**

When we examine the distribution of the Hif-1α binding sites with respect to the nearest transcriptional start site (TSS) we see that 30 % of them fall within the TSS and 6 % fall within the promoter region but that they may also fall at greater distances (Fig. 5). The data detailing the positions of the ChIP peaks can be found in: Additional file 4 - unfiltered ChIP sequencing peaks for *vhl* mutants, Additional file 5 – *vhl* ChIP sequencing peaks filtered for presence of a HRE, Additional file 6 – unfiltered ChIP sequencing peaks for wild-type, and Additional file 7 – wild-type ChIP sequencing peaks filtered for presence of a HRE.

### Validation of ChIP-seq peaks

To validate the ChIP-seq peaks in *vhl*, 20 highly significant peaks containing a HRE, were selected and ChIP-qPCR was performed using primers designed around the region containing the HRE. As negative controls, distant regions which did not contain a peak were selected. qPCR was performed using input and ChIP material and enrichment for the region of interest was determined using a variation on the Pfaffl method as described earlier. In samples from the *vhl* ChIP, all 20 regions showed enrichment of over twenty fold regardless of which control region was used. Additional file 8 shows the fold change values which represent peak enrichment, using the least significant control region, approximately 1Kb down-stream of *jmjd6*. In wild-type ChIP materials, a degree of enrichment was seen for many peaks, which indicates there may be a low level of Hif-1α binding. This is put into context by comparing the ratio of fold change between *vhl* and wild-type. In all but one case *vhl* was seen to be more strongly enriched often by more than 100 fold. With these findings, alongside the observation of the strong representation of HRE’s proximal to the peak summit, we are satisfied that the peaks found in *vhl* mutant ChIP-seq represent Hif-1α binding.

### Comparison of microarray and ChIP-seq data

In order to investigate the extent to which Hif-1α binding influences the transcriptional profile of *vhl* mutant zebrafish, we compared the gene expression changes in *vhl vs.* WT and the peaks for the Hif-1α ChIP performed using *vhl* mutant embryos.

As we found such a high number of Hif-1α binding sites in *vhl* mutant samples (5177), we were concerned that this could lead to a high number of false associations with genes in their vicinity. In order to minimise this, we chose to examine region-gene associations where the peak is within ±1Kb of the TSS. We achieved this by mapping the region-gene associations using Genomic Region Enrichment Analysis Tool (GREAT) selecting for the two nearest genes, then selecting genes within ±1Kb of the TSS [65]. This found 1722 gene associations from 1670 of the peaks that fall within ±1Kb of the TSS. We examined the degree of overlap between the microarray data and the genes associated with Hif-1α peaks in their TSS. We found that of the 295 up-regulated genes (FC ≥ 2), 73 of these were associated with Hif-1α peaks within ±1Kb of their TSS. Whilst of the 442 down-regulated genes, 26 were associated Hif-1α binding peaks within ±1Kb of their TSS. In order to assess the significance of the intersection between the differentially regulated genes in the microarray and the genes associated with Hif-1α DNA binding ±1Kb of their TSS, we performed hypergeometric analysis. Our microarray contained 13691 genes with a unique gene symbol, when GREAT was used to assess the number of genes with Hif-1α DNA binding ±1Kb of their TSS, 1722 genes were found, with 1314 of these being included in the microarray. When the probability that the up-regulated genes (295) associated with ChIP-seq peaks in *vhl* mutants (73) occurred by chance was assessed, hypergeometric testing gave a probability of ≤1.5 10^−14^. When the probability that down-regulated genes (442) associated with ChIP-seq peaks in *vhl* mutants (26) occurred by chance was assessed, a hypergeometric testing gave a probability of ≤0.99. Additional file 9 shows the genes and the peaks associated with them.

Overall, if a gene is up-regulated, the chance that it contains a HRE-peak near the TSS is about four times higher than if it is down-regulated and about 2.5 times more than expected by chance. However, the probability of whether a ChIP seq peak contains an HRE is not related to whether it is associated with an up- or down- regulated gene. Intriguingly, non-HRE peaks show similar enrichment in TSS regions and are found to be associated with up-regulated genes. For instance, 39 % (367/945; non-HRE) and 34 % (1457/4232; HRE) of the peaks were closely associated with TSS regions.

### Comparison with published HRE data in zebrafish and human

In zebrafish HREs are known for *leg1a/b*, *birC5a/b*, *period1b*, *epo* and *igfbp1a*. We confirmed peaks at the predicted position for *igfbp1a*, *epo*, and peaks coinciding with HREs E2 and E3 from *period1b* (but not E4). For *period1b* we found a novel peak in intron1. We were unable to detect the HREs described for *leg1* and *birC5* genes. We have not yet checked whether there are “sub-threshold” peaks at their predicted position in our data set.

To evaluate how well our data corresponds with mammalian HRE data, we did several comparisons. First we asked whether a set of well-known HIF targets had associated peaks in our data. We collated an extensive list of 107 targets based on [66]. In 94 cases we were able to identify a clear orthologue (or orthologue-pair, as zebrafish have undergone a further partial genome duplication) and in 66 of 128 orthologues (51 %) a peak could be identified within the orthologous gene, or the 15Kb surrounding genomic sequence. For a random set of 105 genes this was 32 (30 %), showing significant enrichment (χ2 *p* < 0.002; Additional file 10).

Secondly, for 12 well-known human HIF targets with experimentally characterised HIF-binding sites, we compared the position of the known HIF-binding site to our peaks. We estimated that in roughly 7–9 out of 12 genes, peaks were identified in comparable places near zebrafish orthologues (Additional file 10). Often, we identified further peaks that were not described. We also compared our data with a human ChIP seq dataset [33]. This study identified the largest number of Hif-binding sites until now (but it was also incomplete, as “classical” targets like *EPO*, *VEGF* and *EGLN1* were not found, whereas they are present in our dataset). We analysed more than 50 of their top peaks and analysed whether peaks occurred in similar positions in our study. We only selected peaks that fell within those genes, or <10Kb from TSS/3′UTR. In 15/39 cases similarly positioned peaks were noted (Additional file 10). Sequence comparison of 10 such coinciding peaks in fish and human, failed to uncover significant sequence homologies.

Finally, we used the GREAT website [67] to select all peaks from Schödel et al., that fall within the TSS ± 1Kb from a gene and identified 112 genes. Of these, 102 had zebrafish orthologues. Using our ChIP-seq data, GREAT identified 1722 peak associated genes from 17059 genes with the same characteristics. When the peak associated genes from Schödel et al., were compared with our peak associated genes; out of 102 genes, 25 fell within the set of 1722 (Additional file 10), which represents a significant enrichment (*p* = 2.5 10^−5^) when hypergeometric analysis was performed.

In the zebrafish *pfkfb3* gene, an unusual pattern of peaks was found. *Pfkfb3* is an important regulator of glycolytic flux and part of a family of 4 *pfkfb* genes in mammals that probably arose through genome duplications, as primitive chordates and *Drosophila* only contain a single *pfkfb* gene. Surprisingly, nine peaks were identified near *pfkfb3*. This gene was the only gene in the genome where we found multiple peaks located in the middle of exons: 4 in coding exons and one in the 3′UTR. The peaks in coding exon 8 and the 3′UTR had a (bidirectional) HIF-binding sequence “CACGTG” near the summit. The exon 8 peak was also found in our wild-type sample (Additional file 11). We explored how the HIF binding site in exon 8 evolved. In this unique situation flanking coding sequence conservation can help to follow the binding site over long evolutionary distances. In human, both *pfkfb3* and *pfkfb4* are regulated by hypoxia and both contain the CACGTG sequence. Zebrafish *pfkfb*4*a/b* lack the CACGTG sequence, and, as predicted, do not show exonic ChIP-seq peaks. Other *pfkfb* orthologues in zebrafish lack the CACGTG sequence and also lack ChIP-seq peaks in exon 8 (despite the fact that some retain a “reverse” CACGT consensus sequence). In human, *pfkfb1* and *pfkfb2* also lack the sequence. This element has most likely arisen during early vertebrate evolution, it can first be recognised in a jawless vertebrate, *Pteromyzon marinum* (www.ensembl.org), but not in more primitive chordates or *Drosophila*.

### Discussion

Here we describe the use of ChIP coupled to next-generation high-throughput sequencing to identify Hif-1α binding sites throughout the zebrafish genome in the *vhl* mutant. ChIP allows unbiased genome-wide coverage of the zebrafish genome to identify Hif-1α binding sites, enabling development of zebrafish as an emergent model organism for research into the hypoxic response in a physiological context [41, 42, 46, 52, 68]. The use of the *vhl* mutant, a zebrafish model for hypoxia, has allowed us to compare Hif-1α binding sites with gene expression changes seen in this mutant.

Microarray analysis of gene expression changes in *vhl* mutants compared against wild-type controls produced both expected and unexpected results. The up-regulated genes included many classical hypoxia response genes that are associated with loss of *Vhl* in mammalian cell lines (Additional file 1). This reinforces the idea that the hypoxic response is activated in the *vhl* mutant. Contrary to this, we see similar numbers of down-regulated genes. Currently, there is little evidence in the literature for HIF to act as transcriptional repressor. However, pVHL is thought to have both HIF-dependent and HIF–independent functions [69]. The latter include; extracellular matrix deposition [70], cell senescence [71], apoptosis [72] and microtubule stabilisation and regulation of the primary cilia [73]. It is not inconceivable that loss of Vhl’s HIF-independent functions may lead to a significant number of both up- and down-stream transcriptional changes. Furthermore, these transcriptional changes may be complicated by the use of a whole organism model. *Vhl* mutants have had activation of the hypoxic response over a period of several days, leading to additional transcriptional changes which would not be identified in cell-culture models, using more acute stimulation. Interestingly, we see that of these down-regulated genes, 12 are predicted to be involved in ‘lipid biosynthetic processing’ by DAVID (Additional file 3). This finding is of interest as HIF-2 activation was reported to affect lipid metabolism in hepatocytes causing a decrease in lipogenic gene expression and on a physiological level, leading to hepatic steatosis in liver-specific Hif-2α mutant mice [21]. Although the histology of the liver has not been reported in *vhl* mutant zebrafish, evidence of increased lipid storage has been detected in the pronephric tubules of *vhl* mutant embryos [44]. The effects of hypoxia on lipid processing have not been well studied, and strong representation of genes related to this process in the *vhl* mutant, in our view, warrants further study. This highlights an important consequence of using whole zebrafish to study the effects of hypoxia. The presence of a variety of cells, tissues and organs in a whole animal may on the one hand complicate analysis, however, for the same reasons novel, more tissue-specific HIF targets can be found. In addition, targets might be found whose expression is modulated indirectly, as a result of signalling between tissues in the intact organism.

To locate Hif-1α binding sites we first identified the most highly represented motif found within the peaks seen in *vhl* mutants. An RCGTG (with preference for A) motif that is identical to the consensus motif identified in human hypoxia response genes was found using genome-wide ChIP approaches (Fig. 2) [33, 34]. Additionally, it is highly similar to the HRE identified from the Hif binding regions of *epo* in another fish species *Takifugu rubripes* [35]. This is both reassuring and unsurprising as the HIF pathway is strongly conserved throughout the phylogeny [74].

Our experiments identified 4323 candidate HIF binding loci and possibly up to 5177 sites, a greater number of loci than might have been expected, as previous studies have identified approximately 400 HIF-1α binding loci in human cells [33]. Despite this, we are confident that our 4323 sites are due to Hif-1α binding. Firstly, the majority were not identified in WT animals where nuclear Hif-1a levels are expected to be very low, thus they are unlikely to pulled down due to spurious interactions of the antiserum and unrelated DNA binding proteins. Secondly, they contain the highly conserved RCGTG in close proximity to the peak summit (Fig. 4). We interpret this as a strong indicator that these HRE-containing peaks are due to Hif-1α binding.

It is notable that 945 of the peaks we found do not contain RCGTG motifs in the surrounding 100 bp, the relevance of these peaks is currently unknown. We investigated whether these non-HRE peaks were due to non-specific binding of the anti-Hif-1α antibody, this was achieved by examining the overlap between these peaks with all the peaks found in wild-type embryos (1284). We found that, although 45 of the 945 peaks found in *vhl* mutants overlapped >50 % with the wild-type peaks (Additional file 12), the vast majority of these peaks did not overlap. We reason that if the anti-Hif-1α antibody is binding non-specific DNA-interacting targets, then this would lead to non-HRE containing ChIP peaks. However, we would also predict that many of these peaks would be shared between wild-type and *vhl* samples. As we find this is not the case for the majority of ChIP peaks, and that MEME analysis did not detect any other motifs, we suggest that there may be other explanations for the non-HRE peaks. These could represent HIF binding to off-centre HREs or indirect binding via other DNA binding proteins or alternatively, these peaks may contain HREs that do not conform to the RCGTG consensus. We were interested if there were indications that these non-HRE peaks may represent Hif-1α binding leading to changes in gene expression. Using GREAT, we compared the distribution of non-HRE peaks with that of HRE-peaks and found a very similar pattern. Although this association does not prove the functional importance of these non-HRE peaks, it suggests that they may have a role in regulating transcription.

Although we do not have functional data to draw direct links between Hif-1α DNA binding and transcriptional activation we were interested to see if Hif-1α peaks in the TSS correlated well with gene expression changes in *vhl* mutants. In *vhl* mutants, we found that of the 295 up-regulated genes, 73 had Hif-1α peaks in their TSS regions whilst of the 442 down-regulated, 26 had Hif-1α peaks in their TSS regions. Although we found some Hif-1α peaks associated with down-regulation of gene expression, we found a much larger proportion associated with transcriptional activation. When we performed hypergeometric analysis of these data sets, we found that the correlation between up-regulated genes and Hif-1α binding peaks was significant (*p* ≤ 1.5 10^−14^), whilst it was not for down-regulated peaks (*p* = 0.99). This is broadly in agreement with the consensus in the literature, that Hif-1α is not thought to directly regulate transcriptional repression [75], but functional experiments to investigate the possibility direct Hif-1α mediated repression of gene expression would be of interest. It should however also be noted that many of the genes up-regulated in *vhl* microarrays did not have HRE’s in their TSS regions. It is as yet unclear as to why this is exactly. Importantly, our analysis of the relationship between Hif-1α binding and gene expression has focused on peaks that fall within the TSS, however, many peaks are found outside the TSS and promoter regions (Fig. 5), which is in agreement with the situation in human [33]. Unfortunately these peaks are very difficult to assign reliably to genes, as distance to the TSS can be misleading to detect functional enhancers. In addition, as HIF is a master regulator and activates other signalling pathways (eg VEGF, Epo) and transcription factors, indirect targets are undoubtedly present in the microarray.

Our study found a much higher number of significant peaks in *vhl* mutant zebrafish than have previously been found in cell culture studies where physical hypoxia or DMOG have been used to stimulate the HIF response [33, 34]. Indeed, we find a considerably higher number of significant peaks containing the RCGTG motif than we find up-regulated genes. This begs the question: why are there so many more peaks found using the *vhl* mutant zebrafish than hypoxically stimulated cells? A number of explanations are conceivable. Firstly, the embryos we used at 4dpf and have been stimulated for a longer time than the cells used by Schödel et al., who expose them for 16 h, this could lead to greater accumulation of Hif-1α [33]. Secondly, the *vhl* zebrafish contains a wide array of cells and tissues which may have different Hif-1α DNA binding profiles, thus leading to a greater number of peaks than would be found from just one cell-type. Finally, we have used a genetic method to stimulate the Hif pathway, which blocks the degradation of Hif. We speculate that this leads to a glut of Hif-1α accumulating in the nucleus, and thus promoting more binding than would be seen by physiological activation [76]. In order to ascertain whether individual peaks are functional, further work is needed.

Our data can be used confirm known and identify novel potential HIF regulated genes, including, for example, miRNAs. Some examples of this are given in Additional file 11. In zebrafish Hif-target genes, we can predict where HRE are located to guide further experiments. In addition, we see some conservation of where HRE peaks are located when compared to human, so our data may also guide HRE discovery in mammals. For example, we predict an important HRE to be present in exon 8 of the human *pfkfb3* and *pfkfb4* genes.

## Conclusions

Here we present transcriptional analysis of the *vhl* mutant with respect to Hif-1α as a known regulator of the hypoxic response. We found that, unlike some cellular models, which use physical hypoxia or pharmaceutical stimulation to inhibit the PHD’s, genetic inactivation of *vhl* led to both positive and negative transcriptional changes. This may be due to disruption of Vhl’s Hif-independent roles, or alternatively this may be due to studying the effects of activation of genetically induced hypoxia in a whole organism. ChIP-seq for Hif-1α in *vhl* mutants found a great number of peaks and from these the canonical HRE was identified. We found more HRE-containing peaks than transcriptionally activated genes, suggesting that Hif-1α binding may not be sufficient for transcriptional activation. Some ChIP peaks did not contain a HRE, although they do not appear to be due to non-specific binding of the anti-Hif-1α antibody and some are found within the TSS regions of transcriptionally active genes suggesting that they may have some functional relevance. We hope that these data can be used to aid further investigation into the Hif transcriptional response and the development of the zebrafish as a model for research into the hypoxic response.

## Methods

### Zebrafish husbandry and lines

Fish husbandry was performed at the Bateson Centre aquaria at The University of Sheffield and conformed to UK Home Office requirements. Breeding and experiments were approved by the ethics committee in the University of Sheffield and performed under project licence numbers which were 40/3262, 40/3082 and 40/3641 issued to Dr Freek van Eeden and under the personal license 40/9657 issued to David Greenald. Adult fishes were maintained on a 14:10-h light/dark cycle at 28 °C with feeding of live artemia and/or dry food twice daily.

The Tg(*phd3::EGFP*)^i144/i144^ line is a hypoxia reporter line created by Bacterial Artificial Chromosome-mediated transgenesis [46]. The *vhl*^*hu2117/+*^line was crossed into the Tg(*phd3::EGFP*)^i144/i144^ line in order to create *vhl*^*hu2117/+*^*;phd3:gfp*^*i144/i144*^ fish. When incrossed these fish produce *vhl*^*hu2117/hu2117*^*;phd3:gfp*^*i144/i144*^ embryos in Mendelian ratios [46]. Fish were pair mated and embryos were sorted into groups of 40 and placed in Petri dishes containing fresh E3 (5 mM NaCl, 0.17 mM KCl, 0.33 mM CaCl_2_, 0.33 mM MgCl_2_, pH 7.2) media containing methylene blue (Sigma-Aldrich) at 0.0001 % and raised at 28 °C.

### RNA isolation, processing and microarray hybridisation

Three batches of 30 4dpf wild-type and *vhl* mutant embryos were snap-frozen and stored at −80 °C. Extraction of total RNA from zebrafish embryos was performed using the mIRVANA miRNA isolation kit (Invitrogen) excluding the step for enrichment of miRNA. Extracted RNA was quantified using a Nanodrop ND-1000 spectrophotometer. Quality of RNA was assessed using Agilent Bioanalyser 2100 Nanochip 6000, samples with RNA Integrity Numbers ≥ 7 were accepted.

Microarrays utilised custom designed oligonucleotide arrays designed by Compugen and synthesised by Sigma-Genosys [77]. Microarrays were performed on the Agilent platform using one-colour microarray-based gene expression analysis using Cy3-labelled targets to measure gene expression. Using 400 ng RNA as starting material, Cy-3 labelling, hybridisation, washing, scanning and feature extraction were performed according to manufacturer’s protocol (One-Colour Microarray-Based Gene Expression Analysis (Quick Amp Labeling), Version 5.7, March 2008) for 4 x 44 K microarrays. cRNA was quantified using the Nanodrop ND-1000from which yield and labeling efficiency were calculated. The quality of cRNA was measured using an Agilent Bioanalyser 2100 with Nanochip 6000 to check that the majority of the cRNA falls within 200–2000 bp and that there were no distinct bands below 200 bp.

Scanning was performed using the GenePix 4000B (Axon Instruments) according to manufacturer’s protocol (One-Colour Microarray-Based Gene Expression Analysis (Quick Amp Labelling), Version 5.7, March 2008) producing a 16-bit TIFF image upon which feature extraction was applied using Aglient Feature Extraction software version 10.7.3.1.

### Custom oligonucleotide zebrafish arrays (Agilent)

The array chips contain 41,091 probes, which corresponded to 28864 unique entities with a Unigene ID when the custom annotation software (GIS Unigene and Gene Ontology Annotation tool, http://123.136.65.67) was employed. Notably, these Unigene ID’s corresponded to 110649 fully annotated genes, i.e. those which have a Gene Symbol and Gene description, and 14321 of which corresponded to transcribed loci/CDNA clones and partially annotated genes.

### Microarray data analysis

Quality of microarray data was assessed using the ‘Quality Control on Samples’ function using Genespringf 12.6 GX (Agilent). Limma Version 3.24.10 (Biocondutor) was used to load three samples from raw text files for both *wild-type* and *vhl* mutants. Background correction was applied using the “normexp” function and normalisation was performed using normalise between arrays “quantile” function. To average replicate spots, the “avereps” function was applied. The linear model was applied using “lmfit” and a contrast matrix was applied using eBayes (fit2). From these normalised entity lists, a significance cut off of *p* ≤ 0.01 and fold change cut off of ≥ 2 were applied.

To annotate the Probe ID’s, probenames are exported to the GIS Unigene & Gene Ontology Annotation Tool (http://123.136.65.67/) and the corresponding Genbank Accession and Unigene ID’s were found for *Danio rerio* (DR Build #126_66).

### Microarray validation

The degree of concordance between the fold change values from the microarray analysis and the qPCR was calculated using Spearman’s coefficient [61]. cDNA was created from the same pool of RNA used for the microarray experiments by reverse transcription using Superscript III First-Strand Synthesis System (Invitrogen) using 1 μg RNA and OligiodT. qPCR for Microarray Validation carried out using the Applied Biosystems Sequence Detection System (SDS) Software v2.4.1 in conjunction with 7900HT Fast Real-Time qPCR System.

qPCR was carried out in a 384 well plate format, using the Applied Biosystems Sequence Detection System (SDS) Software v2.4.1 in conjunction with 7900HT Fast Real-Time qPCR System. Cycling conditions of 50 °C - 2 min, 95 °C - 10 min, [95 °C – 15 s, 60 °C - 1 min] × 40 cycles were used. Cycle threshold (Ct) values were calculated automatically using the software, with ROX used as the passive reference.

cDNA concentration curves were created using 4dpf WT RNA, starting at 500, 150, 50, 15, 5, 1.5, 0.5 and finally 0 ng/μl. Primers were tested in triplicate, and efficiencies were calculated from averaged Ct values plotted against the log_10_ of concentration of cDNA and the gradient (m) line of best fit was taken. Efficiency was calculated from the average of 3 technical replicates for each primer.

$$ \mathrm{Efficiency}={10}^{\left(\hbox{-} 1/\mathrm{m}\right)} $$

$$ \mathrm{F}\mathrm{C}=\left({\mathrm{E}}_{\mathrm{t}\mathrm{ar}}{}^{\mathrm{d}}{}^{\mathrm{C}}{{}^{\mathrm{t}}}_{{}^{\mathrm{t}}{}^{\mathrm{a}}{}^{\mathrm{r}}}\right)/\left({\mathrm{E}}_{\mathrm{C}\mathrm{N}}\kern0.333em {}^{\mathrm{d}}{}^{\mathrm{C}}{{}^{\mathrm{t}}}_{{}^{\mathrm{C}}{}^{\mathrm{N}}}\right) $$

This was performed for *vhl vs* wild-type.

The suitability of the reference gene was then assessed by performing Kruskal-Wallis One-way ANOVA with Dunn’s Multiple testing (*P* < 0.05) for each of the above comparisons. *Wnt5a and* 18 s passed for all comparisons and were subsequently used for Microarray Validation. Additional file 13 contains the details of the primers used for microarray validation.

### ChIP-sequencing: sample preparation

*Vhl*^*hu2117/+*^*;phd3:gfp*^*i144/+*^ fish were incrossed and *Vhl*^*hu2117hu2117+*^*;phd3:gfp*^*i144/+*^embryos were sorted from non-fluorescent siblings prior to de-yolking. *Vhl* mutants were sorted and deyolked by trituration in 4 °C deyolking buffer (55nM NaCl, 1.8 mM KCl, 1.25 mM NAHCO_3_, 1x Complete protease inhibitor (Roche) ) followed by shaking on a bench-top vortex at 1000 rpm for 5 min. Embryos were pelleted by centrifugation at 1000 rpm for 30 s at 4 °C and supernatant discarded. Embryos were washed using wash buffer (100 mM NaCl, 3.5 mM KCl, 2.7 mM CaCl_2_, 10 mM Tris–HCl pH 8.5, 1x Complete protease inhibitor (Roche)) with 2 min shaking at 1000 rpm on a bench-top vortex. Embryos were washed in 4 °C 1X PBS and pelleted by centrifugation at 1800 rpm. Embryos were then macerated and resuspended in 10 ml 1.1 % formaldehyde in 1 X PBS (5 mM Hepes-KOH pH 7.5, 10 mM NaCl, 100 μM EDTA, 50 μM EGTA, 1.1 % formaldehyde, 1x Complete protease inhibitor (Roche)). The embryos were incubated for 10 min at room temperature, gently rocking. Formaldehyde was quenched by addition of 1 ml of 2.5 M glycine with 1x Complete protease inhibitor (Roche) and incubation for 5 min with gentle rocking. Samples were then spun at 3500 rpm at 4 °C for 5 min in an Eppendorf 5810 R Centrifuge before snap freezing in liquid nitrogen. Once 2400 embryos were collected for wild-type and for *vhl* mutants, ChIP could proceed.

### ChIP-sequencing: cell sonication

Macerated embryos were lysed in 3 ml Cell Lysis Buffer (10 mM Tris–HCl pH 7.5, 10 mM NaCl, 0.5 % Igepal CA-630, 1 x Complete protease inhibitor (Roche) and incubated on ice for 15 min. Samples were centrifuged at 3500 rpm and the supernatant discarded. The pellet was resuspended in 1.5 ml Nuclei Lysis Buffer (50 mM Tris–HCl pH 7.5, 10 mM EDTA, 1 % SDS and 1X Complete protease inhibitor (Roche)) and incubated over ice for 10 min. Two volumes of Immunoprecipitation Dilution Buffer (16.7 mM Tris–HCl pH 7.5, 167 mM NaCl, 1.2 mM EDTA, 0.01 % SDS, 1x Complete protease inhibitor (Roche)) were added and the samples were sonicated at 25 cycles at 40 % amplitude, 18 s pulse and 59.9 s rest using a Branson Sonicator 450D. After sonication, 300 μl 10 % Triton was added to 4 ml chromatin followed by centrifuged at 13,300 rpm for 10 min at 4 °C and the supernatant was saved. 150 μl of the cell lysate was saved at −20 °C to create input control.

### ChIP-sequencing: pre-block and binding of Hif-1α antibody to magnetic beads

100 μl Dynabeads A magnetic beads (Life Technologies) were washed with 1 ml PBS with 1 X Bovine Serum Albumin. Beads were collected by centrifugation at 3000 rpm for 3 min at 4 °C in 1.5 ml tubes. The beads were washed 2 x in 1 ml PBS-BSA and collected with a magnet. 10 μg of Hif-1α antibody in 250 μl PBS/BSA was added to the beads and incubated overnight at 4 °C. The beads were then washed 3 x in 1 ml PBS-BSA and resuspended in 100 μl PBS/BSA.

### ChIP-sequencing: chromatin immunoprecipitation

The pre-blocked Dynabeads were added to the sonicated chromatin and incubated overnight at 4 °C.

### ChIP-sequencing: wash, elution and cross-link reversal

Beads were precipitated using a magnetic stand and washed x8 in 4 °C RIPA wash buffer (50 mM Hepes-KOH pH 7.6, 1 mM EDTA, 500 mM LiCl, 0.7 % Na-Deoxycholate, 1 % NP-40), x1 in TBS and centrifuged for 3 min at 3000 rpm after which all TBS was aspirated. 250 μl of Elution Buffer (50 mM Tris–HCl pH 8.0, 10 mM EDTA, 1 % SDS) was added prior to incubation at 65 °C for 15 min in a water bath with brief vortexing every 2 min. The beads were centrifuged at 13,300 rpm for 1 min and the supernatant saved as this contains the ChIP material.

The input sample saved earlier was thawed and 3 volumes of Elution Buffer was added. Both ChIP and Input samples were incubated at 68 °C for 3 h shaking at 1300 rpm to reverse cross-linking.

### ChIP-sequencing: digestion of cellular protein and RNA

Three times volume TE was added to each sample, RNaseA (0.2 μg/ml final concentration, Qiagen) was added and samples were incubated at 37 °C for 30 min to degrade RNA. To degrade protein, proteinase K (Sigma-Aldrich) (0.2 μg/ml final concentration) was added and incubated at 55 °C for 1 h.

To isolate DNA, 400 μl phenol:choloform:isoamylalcohol (Ambion) was added to each sample and phases separated using 2 ml Heavy Phaselock (Qiagen) according to manufacturer’s instructions. The aqueous layer was recovered and NaCl (200 mM final concentration) and 30 μg glycogen from *Mytilus edulis* (Sigma-Aldrich) was added. 800 μl ethanol was added and the samples were incubated for 1 h at −80 °C prior to centrifugation at 13,300 rpm at 4 °C for 10 min. Supernatant was removed, and the pellet washed with 75 % Ethanol, before being air-dried and resuspended in 20ul 10 mM Tris–HCl pH 8.5. DNA concentration was measured using the QuBit 2.0 system (Invitrogen).

### ChIP-sequencing: ChIP-qPCR

Primers were designed around the EPO-HRE, see Additional file 14 for details.

Primers were validated using a DNA concentration curve of 300, 100, 30, 10, 3, 1 and 0 ng genomic DNA from zebrafish. qPCR was performed using the 7500 Applied Biosystems qPCR Real-time PCR system using the cycling conditions: 50 °C - 2 min, 95 °C - 10 min, [95 °C – 15 s, 60 °C - 1 min] x 40 cycles, with the additional pre-programmed dissociation stage. Primers were tested in triplicate and efficiencies were calculated from averaged Ct values plotted against the log_10_ of concentration of DNA and the gradient (m) line of best fit was taken. Efficiency was calculated from the average of 3 technical replicates for each primer.

$$ \mathrm{Efficiency}=10{}^{\left(\hbox{-} 1/\mathrm{m}\right)} $$

qPCR was performed using 4 samples for each primer set, wild-type input and ChIP and *vhl* mutant and ChIP. For each qPCR experiment only 0.01 ng of DNA was used. Fold change was calculated using a variation on the Pfaffl ratio [58]. The change in Ct value between Input and ChIP for both the target region (dCt_tar_) and the reference region (dCt_ref_) were calculated thus:

$$ \mathrm{d}\mathrm{C}\mathrm{t}={\mathrm{Ct}}_{\mathrm{Input}}\hbox{-} {\mathrm{Ct}}_{\mathrm{ChiP}} $$

$$ \mathrm{F}\mathrm{C}=\left({\mathrm{E}}_{\mathrm{t}\mathrm{ar}}{}^{\mathrm{d}}{}^{\mathrm{C}}{{}^{\mathrm{t}}}_{{}^{\mathrm{t}}{}^{\mathrm{a}}{}^{\mathrm{r}}}\right)/\left({\mathrm{E}}_{\mathrm{C}\mathrm{N}}{}^{\mathrm{d}}{}^{\mathrm{C}}{{}^{\mathrm{t}}}_{{}^{\mathrm{C}}{}^{\mathrm{N}}}\right) $$

Additional file 15 contains details of the primers used to validate the sequencing.

### ChIP-sequencing: library preparation

Using 5 ng of ChIP material and 10 ng of input material for both wild-type and *vhl* mutants library preparation was performed using the Illumina Truseq ChIP Sample Preparation Kit set A (REF: 15034288) in accordance with manufacturer’s instructions using the adapters 2, 7 for input in duplicate and 19 for the ChIP samples to allow multiplexing. Following library preparation, the amplified adapter bound materials were tested for size and approximate quantification using High Sensitivity DNA assay using the 2100 Agilent Bioanalyser according to manufacturer’s instructions. On the basis of the approximate quantification, precise quantification was performed using qPCR as recommended by the protocol following internal guidelines for submission of sequencing samples at the Genome Institute of Singapore. Samples of 8pM, 4pM, 2pM and 1pM were amplified using appropriate adapter primers at 0.1 μM and Roche SYBR Green I Master in a Roche Lightcycler 480. Cycling conditions of pre-incubation at 95 °C for 5 min followed by 30 cycles of 95 °C for 10s sec, 60 °C for 1 min, and 72 °C for 30 s, then a melting curve 95 °C 5 s, 65 °C for 1 min and 97 °C for 10 s decreasing 5 °C each increment before cooling to 40 °C. Quantification is achieved by exporting crossing point values and plotting them against log concentration and performing linear regression to calculate the line of best fit to be used in y = mx + c where and using this to calculate the concentration. 15 μl of 5nM between 200–300 bp is submitted for sequencing.

### ChIP-sequencing: sequencing

Sequencing was carried out a Genome Institute of Singapore using the Illumina Genome Analyser II (Illunima, USA) performing a single read with 3 samples per lane to allow multiplexing. Output files were analysed by Dr. Justin Jeyakani at GIS. Sequencing runs for WT input material produced 34,915,101 unfiltered reads, 32,602,307 which passed filtering and 19,400,899 reads which mapped uniquely using CASAVA (v 1.8.2, Illumina). For the WT ChIP Material, there were 31,424,233 total reads, 30331510 of which passed filtering and 16,780,814 reads which mapped to uniquely. For the VHL sequencing, input produced 24,823,207 total reads, 23,325,383 of which passed filtering, and 13,834,669 which mapped uniquely. The VHL ChIP material produced 65,797,779 total reads, 63,035,293 which passed filtering and 33,429,235 which mapped uniquely. Only reads which mapped uniquely were used for peak calling. All reads were 35 bp in length.

Peak calling was performed comparing ChIP material to input using MACS (version 1.4.2 20120305), the binding regions were ranked based on the enrichment of sequencing tags by comparing each ChIP library to control [78]. A threshold of *p*-value > 1 × 10^−5^ was applied, this gave rise to 5177 peaks in *vhl* ChIP and in wild-type ChIP material versus wild-type input 1280 peaks were called. Alignment was carried out using CASAVA (v 1.8.2, Illumina) with danRer7-Zv9 as the build.

*De novo* motif analysis was carried out using MEME (version 4.9.0, http://*meme-suite.org* ) using ±50 bp from the summits of the top 1000 peaks [63]. This identified an RCGTG motif (Fig. 2). This motif was then used to filter the peaks for the presence of the motif using MOODS (v 1.0.1, http://www.cs.helsinki.fi/group/pssmfind/) with an e-value cut-off of 0.001 and Zv9 genome as a background, this identified 2736 peaks in *vhl* ChIP samples [64]. However, as this omitted most experimentally confirmed fish HREs, we chose to filter peaks for the presence of a simple RCGTG sequence. In wild-type samples, MEME was not able to find any robust motifs. These wild-type peaks were then scanned for the motif found in the *vhl* ChIP peaks using MOODS which found 50 sites [64], whereas simple RCGTG detection resulted in 168 sites. Peaks were confirmed on the basis of tag distribution.

To show percentage of peaks that contain a HRE motif within 100 bp of the peak, the peaks were ranked on the basis of the MOODS *p*-value and then binned into groups of 1000 i.e. top 1 K, 1-2 K, 2-3 K, 4 K and above (Fig. 3). The percent of peaks that contain a HRE was then calculated for each bin. Similarly, scanning for motif enrichment in the 1Kb region surrounding the peaks to show the proximity of the motifs to the peak. Techniques employed to perform these analyses can be found in [79–81] (Fig. 4).

To assess the genomic regions *vhl* mutant ChIP peaks fell in, distance to TSS was assessed using Genomic Region Enrichment Annotations Tool (GREAT) version 2.02, using the singles nearest gene rule [65]. Regions were then assigned according to the diagram in Fig. 5a. In order to calculate the amount of the zebrafish genome that falls within these categories, a custom script was created, which is available at https://github.com/iansealy/bio-misc/blob/master/classify_genome_bases.pl.

To identify the genes associated with peaks Genomic Region Enrichment Annotations Tool (GREAT) version 2.02 was used [65]. The Wellcome Trust Zv9 (danRer7, Jul 2010) assembly was used with the two nearest genes being used as the association rule, only peaks found with the TSS region (±1 kb from TSS) were used.

### Gene ontology analysis

Gene Ontology Analysis was performed using DAVID Bioinformatics resource 6.7 (http://david.abcc.ncifcrf.gov/home.jsp). Unigene ID lists for up- and down-regulated genes were uploaded separately, using Aglient Zebrafish background and ‘GO TERM BP FAT’ was used to assess functional relevance of gene expression changes [56, 57].

### Comparisons with published human data

A HIF-response gene list was collated based on [66] (Additional file 10). The peak position data from [33] were converted from NCBI36/hg18 to NCBI37/hg19 using http://genome.ucsc.edu/cgi-bin/hgLiftOver and the resulting bed file was analysed using GREAT and the UCSC (http://genome-euro.ucsc.edu) browser. Comparisons were done using our 5177 peak-set. We extracted lists of genes associated with a peak at ±1Kb of the TSS using GREAT for both our and the Schödel dataset, to determine overlap.

### ANOVA

For data analysed using ANOVA, normality of the data was assessed using D’Agostino and Pearson omnibus normality test. If data passed at α = 0.05 then One-Way ANOVA was performed, if not or the n number was too small a Kruskal-Wallis test was performed. For One-Way ANOVA a *P* value of *p* < 0.05 was the measure of significance. For Bonferroni multiple comparison tests used to compare pairs of groups a *p* < 0.05 as taken as significant. If data did not pass D’Agostino Pearson, Kruskal-Wallis ANOVA was performed with *p* < 0.05 taken as significant, Dunn’s multiple comparison test *p* < 0.05 was used to calculate significance between columns. The descriptive statistics tool was used to calculate the mean and standard error of the mean which is shown for bar charts.

### Hypergeometric analysis

Hypergeoemtric analysis was performed using the Geneprof Hypergeometric analysis calculator (https://www.geneprof.org/GeneProf/tools/hypergeometric.jsp). For the intersection between the ChIP-seq and microarray data, for “Population size”, we used the total number of genes in the microarray with a gene symbol, for “Number of successes in the population” we used the number of genes associated with a ChIP peak within ±1Kb of their TSS in *vhl* mutants according to GREAT using the two nearest genes association rule and then filtering for presence in the microarray. The “Sample size” is the number of genes either up- or down-regulated in the microarray (FC ≥ 2, *p* ≤ 0.01), and “Number of successes in the population” is the number of genes either up- or down-regulated in the microarray associated with a ChIP-seq peak ±1kB to their TSS’s in *vhl* mutants*.*

For the intersection between the Schodel et al., and our ChIP-seq data, for “Population size”, we used the total number of genes found in GREAT. For “Number of successes in the population” we used the total number of ChIP-associated peaks from our data found in GREAT (two closest genes, ±1Kb TSS), The “Sample size” is the number of zebrafish orthologues to the ChIP-associated peaks found in Schodel et al.,. Finally, the “Number of successes in the population” is the number is the number of our ChIP-associated genes that have an orthologue in the Schodel et al., data set.

### Supporting data

The data sets supporting the results of this article are included within the article and its additional files. The microarray data and ChIP-seq data have been submitted to NCBI Gene Expression Omnibus (http://www.ncbi.nlm.nih.gov/geo/) under the super series GSE70886. The ChIP-seq data can be found under GSE70727 and the microarray data can be found under GSE70885.

## Additional files

Additional file 1:
**Gene Expression Changes in** ***vhl*** **mutant zebrafish.** The list of fully-annotated genes which were differentially regulated with a fold change ≥ 2 in *vhl* mutants versus wild-type at 4dpf. Contains; Probe ID, Fold Change, Log Fold Change, Unigene Description, Unigene ID and Gene Symbol. (XLSX 65 kb) Additional file 2:
**The unfiltered list of differentially regulated genes for** ***vhl*** **vs** **wild-type.** The unfiltered list of genes which were differentially regulated with a fold change ≥ 2 in *vhl* mutants versus wild-type at 4dpf. Contains; Probe ID, Log 2 Fold Change, AveExpr: Average Expression across all arrays and Channels, t: moderated t statistic, *p*-value, add.j.*p*-value: *p*-value adjusted for multiple testing, B: B statistic, the log of the odds that the probe is differentially expressed, Unigene Description, Unigene ID and Gene Symbol. (XLSX 6344 kb) Additional file 3:
**The complete list of gene ontology terms found by DAVID.** Using data found in Additional file 2, DAVID was used to assign gene ontology to genes using the Agilent zebrafish background and Unigene ID as identifier. Number of genes associate with each term, *p*-value and Benjamini correction are shown. (XLSX 12 kb) Additional file 4:
**Unfiltered ChIP sequencing peaks for** ***vhl*** **mutants.** (BED 116 kb) Additional file 5:
***vhl*** **ChIP sequencing peaks filtered for presence of a HRE.** Filtering performed using MACS. (BED 70 kb) Additional file 6:
**Unfiltered ChIP sequencing peaks for** **wild-type.** (BED 56 kb) Additional file 7:
**Wild-type ChIP sequencing peaks filtered for presence of a HRE.** Filtering performed using MACS. (BED 2 kb) Additional file 8:
**Validation of** **ChIP-seq** **peaks by** **ChIP-qPCR.** Table showing the ChIP-qPCR fold changes for both wild-type and *vhl* mutant ChIP samples and the ratio of *vhl*/wt. (XLSX 10 kb) Additional file 9:
**A table of differentially regulated genes containing a ChIP peaks in their TSS.** A table showing the position of ChIP peaks relative the differentially expressed genes from the *vhl* Vs WT microarray data found using GREAT version 2.02. Fold change, Unigene ID and presence of a HRE are indicated. (XLSX 18 kb) Additional file 10:
**Comparison of zebrafish and Human data on HIF target genes.** Sheet 1: collated list of HIF targets based on [65] with fish orthologues and peak positions. A similar analysis of a random sample of genes is also given, Sheet 2: Analysis of a experimentally verified peaks in well-known targets. Sheet 3: Analysis of overlap of top ChIP-seq peaks from [34], Sheet 4: Genes from Schödel with peaks close to TSS, stating overlap with zebrafish orthologues with peaks close to TSS. (XLSX 39 kb) Additional file 11:
**Examples of** **Hif-1α** **binding in proximity to hypoxia response genes.** In all panels the red vertical bar shows the region of the chromosome that is enlarged in the window below. The introns and exons of known genes in the enlarged region are annotated in dark blue, the small chevrons on the genes show the orientation of transcription. The window shows mapped significant peaks for WT and VHL mutants. Peaks appear as black vertical bars. ‘VHL all peaks’ refers to all the peaks found using MACS in the *vhl* mutant samples, ‘VHL HRE only’ refers to the peaks found in *vhl* mutants containing the RCGTG motif in the surrounding 100 bp and thus show a subset of the black vertical bars of “VHL all peaks”, ‘WT all peaks’ refers to all the peaks found in wild-type samples using MACS, and ‘WT HRE only’ refers to the peaks found in the wild-type sample containing RCGTG motif in the surrounding 100 bp. A: A HRE-containing peak −57 bp upstream of Prolyl 4-hydroxylase α1b (*p4ha1b*), a known hypoxia response gene involved in extracellular matrix remodelling which is found to be up-regulated in the *vhl* mutant by FC 34.0 in the microarray [55]. Given that this peak is highly significant, and in the close vicinity to the TSS of a highly expressed gene, it may represent a functional HRE for this gene. B: Screenshot showing a number of Hif-1α binding sites in both *vhl* mutant and wild-type samples surrounding *egln3*. *Egln3* (*phd3*) is a gene which is seen to be strongly activated in response to hypoxia in zebrafish [49], and is up-regulated with a FC of 151 in our microarrays. In *vhl* mutants there is are two strong HRE-containing peak residing within intron 1. Additionally, there are a number of peaks upstream of the gene. Interestingly, there are a number of peaks found in the wild-type sample, suggesting that there is Hif-1α binding, and possible transcription in normoxic environments. Furthermore, these peaks are not at the same sites as found in *vhl* mutants, suggesting that control of *egln3* expression under normoxic environments may be influenced by more factors that Hif-1α binding alone. It would be interesting to discover if these HRE-containing peaks in *vhl* mutants control *egln3* expression, and to what extent the other peaks and “wild-type peaks” contribute to this. We suspect high Hif-1α binding as there is an extremely high fold change found in the microarray and the high fold enrichment of one of the HREcontaining ChIP peaks (28.6). C: Screenshot showing Hif-1α binding in *vhl* mutants surrounding the gene *tiparp*. The data presented here may also be useful for investigating lesser or previously unknown Hifresponsive genes. For instance, TCDD-inducible poly (ADP-ribose) polymerase (*tiparp*), is a gene not previously associated with the Hif response but seen to be up-regulated in the microarray by FC 3.49 which has multiple Hif-1α peaks in its vicinity, some containing HREs. TIPARP was not recognised as being associated with the hypoxic response by gene ontology software such as DAVID or manual literature searches. Interestingly for *tiparp* a xenopbiotic response element has been defined in mouse in the first half of intron 1 [82]. One of the two HRE containing HIF peaks in zebrafish associated with tiparp also maps to the first half of intron 1. As HRE and XRE consensus sequence are essentially identical, this might in fact represent the same enhancer responding to two different inputs. D: Screenshot showing a Hif-1α binding site found in *vhl* mutants in the vicinity of *mir22a-1* Despite not being included in the microarray analysis, there is evidence of Hif-1α binding in close proximity to three microRNAs involved in the regulation of the hypoxic response. Hif-1a binds in close proximity to mir22a-1, a microRNA that suppresses HIF-1α regulated transcription in human colon cancer cells [83]. We also find sites close to mir210 and mir-132/212 which may be involved in the hypoxic response. E: Screenshot showing Chip-seq peaks near *pfkfb3*, which is unique in the genome, as it has several exonic peaks. It also shows several peaks in the surrounding genomic DNA. (PDF 1480 kb) Additional file 12:
**The overlap between** **non-HRE** **ChIP peaks in** ***vhl*** **mutant and** **wild-type** **samples.** Filtering performed manually using the RCGTG motif. (XLSX 9 kb) Additional file 13:
**Details of primers used to validate microarray.** (XLSX 12 kb) Additional file 14:
**Details of primers used in** **ChIP-qPCR** **for the** ***epo*** **-HRE.** (XLSX 22 kb) Additional file 15:
**Details of primers used in** **ChIP-seq** **validation.** (XLSX 10 kb)

## Abbreviations

Bp: Base pair
ChIP: Chromatin immunoprecipitation
ChIP-seq: Chromatin immunoprecipitation linked next generation sequencing
ccRCC: Clear cell renal cell carcinoma
Ct: Cycle threshold
DAVID: Database for annotation, visualization and integrated discovery
Dpf: Days post-fertilisation
FC: Fold change
FIH: Factor inhibiting HIF
GO: Gene ontology
GREAT: Genomic region enrichment analysis tool
HIF: Hypoxia inducible factor
HRE: Hypoxia response element
MACS: Model-based analysis of ChIP-Seq
MOODS: Motif occurrence detection suite
MEME: Multiple Em for motif elicitation
PHD: Prolyl-hydroxylase domain
q-PCR: Quantitative polymerase chain reaction
TSS: Transcriptional start site
UCSC: University of California Santa Cruz
*vhl*: von Hippel Lindau
