# Supplementary material for: Genome-wide mapping of Hif-1α binding sites in zebrafish
Source: BMC Genomics. 2015 Nov 11;16:923. doi: 10.1186/s12864-015-2169-x (PMC4642629; doi:10.1186/s12864-015-2169-x)
Supplement: Additional file 11: — Examples of Hif-1α binding in proximity to hypoxia response genes. In all panels the red vertical bar shows the region of the chromosome that is enlarged in the window below. The introns and exons of known genes in the enlarged region are annotated in dark blue, the small chevrons on the genes show the orientation of transcription. The window shows mapped significant peaks for WT and VHL mutants. Peaks appear as black vertical bars. ‘VHL all peaks’ refers to all the peaks found using MACS in the vhl mutant samples, ‘VHL HRE only’ refers to the peaks found in vhl mutants containing the RCGTG motif in the surrounding 100 bp and thus show a subset of the black vertical bars of “VHL all peaks”, ‘WT all peaks’ refers to all the peaks found in wild-type samples using MACS, and ‘WT HRE only’ refers to the peaks found in the wild-type sample containing RCGTG motif in the surrounding 100 bp. A: A HRE-containing peak −57 bp upstream of Prolyl 4-hydroxylase α1b (p4ha1b), a known hypoxia response gene involved in extracellular matrix remodelling which is found to be up-regulated in the vhl mutant by FC 34.0 in the microarray [55]. Given that this peak is highly significant, and in the close vicinity to the TSS of a highly expressed gene, it may represent a functional HRE for this gene. B: Screenshot showing a number of Hif-1α binding sites in both vhl mutant and wild-type samples surrounding egln3. Egln3 (phd3) is a gene which is seen to be strongly activated in response to hypoxia in zebrafish [49], and is up-regulated with a FC of 151 in our microarrays. In vhl mutants there is are two strong HRE-containing peak residing within intron 1. Additionally, there are a number of peaks upstream of the gene. Interestingly, there are a number of peaks found in the wild-type sample, suggesting that there is Hif-1α binding, and possible transcription in normoxic environments. Furthermore, these peaks are not at the same sites as found in vhl mutants, suggesting that control of egln3 exp [file 12864_2015_2169_MOESM11_ESM.pdf]

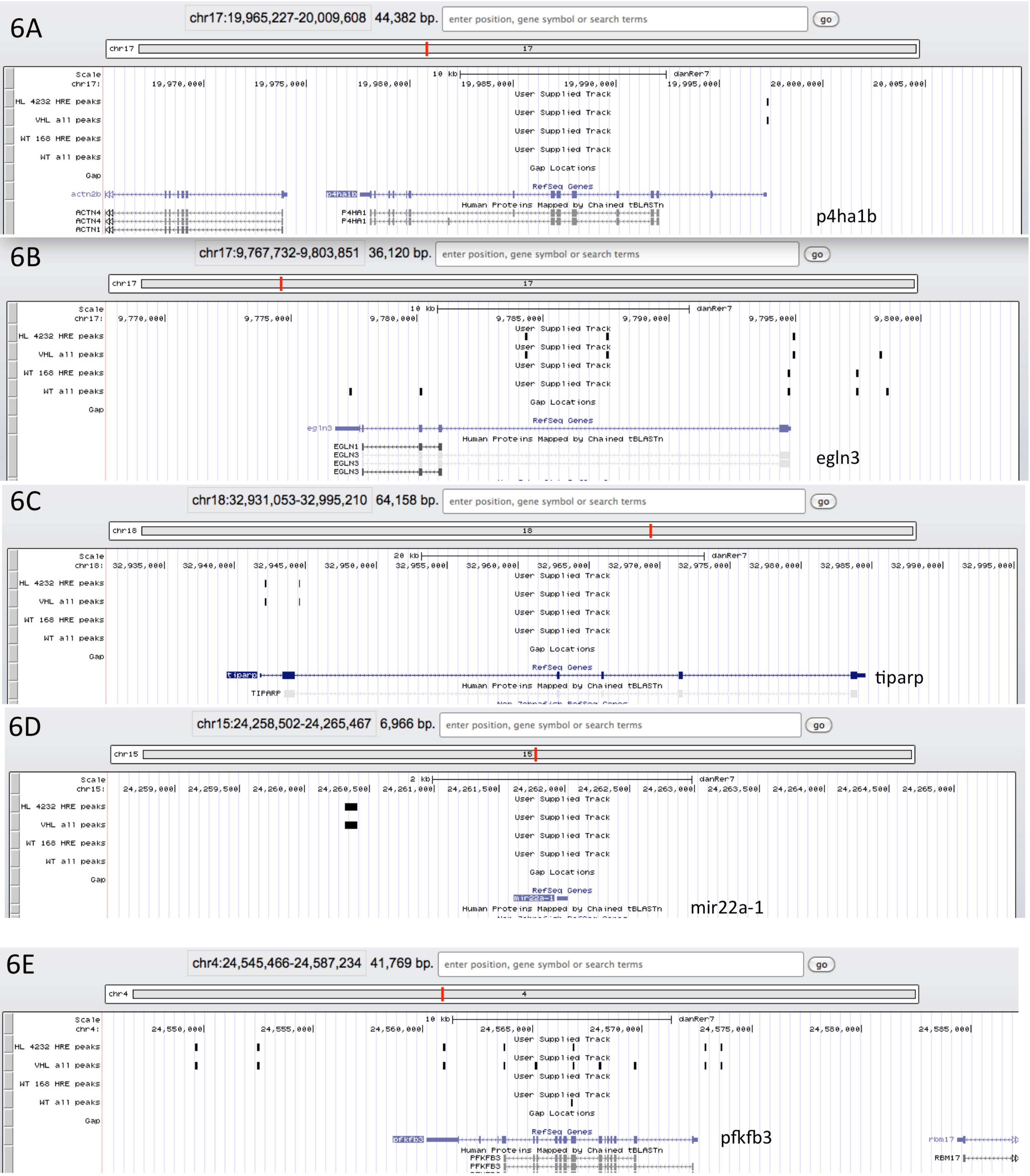
6A
chr17:19,965,227-20,009,608 44,382 bp.
p4ha1b
6B
chr17:9,767,732-9,803,851 36,120 bp.
egln3
6C
chr18:32,931,053-32,995,210 64,158 bp.
tiparp
6D
chr15:24,258,502-24,265,467 6,966 bp.
mir22a-1
6E
chr4:24,545,466-24,587,234 41,769 bp.
pfkfb3
HL 4232 HRE peaks
VHL all peaks
WT 168 HRE peaks
WT all peaks
Gap
RefSeq Genes
Human Proteins Mapped by Chained tBLASTn
